# Exploring the Antibacterial and Antioxidant Effects of *Rhus coriaria* L. Aqueous Extract Against Carbapenem‐Resistant *Acinetobacter baumannii*

**DOI:** 10.1155/ijm/5238068

**Published:** 2026-04-30

**Authors:** Israa Assaf, Ziad Jabbour, Antoine Abou Fayyad, Jamilah Borjac

**Affiliations:** ^1^ Department of Biological Sciences, Beirut Arab University, Debbieh, Lebanon, bau.edu.lb; ^2^ Department of Experimental Pathology, Immunology and Microbiology, American University of Beirut, Beirut, Lebanon, aub.edu.lb; ^3^ Center for Infectious Diseases Research, American University of Beirut, Beirut, Lebanon, aub.edu.lb; ^4^ World Health Organization (WHO) Collaborating Center for Reference and Research on Bacterial Pathogens, Beirut, Lebanon

**Keywords:** *Acinetobacter baumannii*, antibacterial activity, antibiofilm activity, antioxidant activity, CRAB, *Rhus coriaria*

## Abstract

Infections caused by carbapenem‐resistant *Acinetobacter baumannii* (CRAB) are a major concern for clinicians due to its multidrug resistance profile. This has led them to revert to traditional treatment methods. This study is aimed at assessing the antibacterial and antioxidant effects of the aqueous extract of *Rhus coriaria*. Whole‐genome sequencing of CRAB isolates was carried out. The extract was screened for its antioxidant activity. Antibacterial activities were detected by determining the minimum inhibitory and bactericidal concentrations (MIC and MBC), and the time‐dependent growth inhibition assay for the assessment of the extract′s time and dose dependency. The antibiofilm inhibition and destruction activities were also tested using the crystal violet assay, and the ability of the extract to induce hemolysis of erythrocytes through the hemolytic assay. This study proved that *R*. *coriaria* aqueous extract has antioxidant activity reaching 90.69% at a concentration of 3 mg/mL. Moreover, this extract has no toxicity effect on human red blood cells, with a hemolytic activity of 1.3% at this concentration. Additionally, good antibacterial and antibiofilm activities of *R. coriaria* were also detected against all CRAB isolates, with MIC and MBC values of 0.75 mg/mL, and biofilm inhibition reaching 95.22% at 2× MIC. A time‐ and concentration‐dependent effect of the extract was also determined, with a significant rapid decline in growth observed at 6 h of treatment at 2× MIC, then 1× MIC. This study suggests *R. coriaria* extract as an effective adjuvant to antimicrobials for treating multidrug‐resistant bacterial infections.

## 1. Introduction

The global spread of antimicrobial resistance (AMR) poses a serious threat to the world′s public health. Additionally, it not only imposes a significant economic burden on healthcare but also increases morbidity and mortality [1]. Unfortunately, the misuse, overuse, lack of proper antibiotic stewardship, and antibiotic use guidelines, especially in underdeveloped countries, contribute to the decreased effectiveness of antibiotics [2]. *Acinetobacter baumannii (A. baumannii)* is a Gram‐negative coccobacillus, characterized as nonfermenting, nonmotile, nonfastidious, aerobic, oxidase‐negative, and catalase‐positive microorganism [3]. Its infections are associated with bacteremia, pneumonia, meningitis, wounds, and urinary tract infections [4]. Moreover, an increasing pattern of resistance against antimicrobial agents, the slow development of new antimicrobials, and restricted treatment options have led to the increased risk of infection [5]. Carbapenems are a group of antimicrobials used as a last resort to treat infections caused by multidrug‐resistant (MDR) *A. baumannii* strains [6]. This resistance, which has been reported worldwide, is a sign of concern for clinicians due to the burden of infection it causes [5]. Countries in the Mediterranean region, including the Middle East, Southern Europe, and North Africa, have the highest carbapenem resistance rates for *Acinetobacter baumannii* (CRAB), reaching 90%. The United Arab Emirates, Bahrain, Saudi Arabia, Palestine, and Lebanon are the countries in the Middle East with the highest rates of MDR *A. baumannii* infections [7]. The Center for Disease Control (CDC) and the World Health Organization (WHO) classified CRAB as a high‐priority antibiotic‐resistant pathogen [8]. There is widespread concern that CRAB infections will soon become untreatable [2]. Hence, an urgent need for novel, innovative treatments and medications for CRAB infections is a necessity [6]. Plants are recognized to have considerable pharmacological properties, such as antioxidant, antibacterial, anti‐inflammatory, and anticancer capabilities due to their phytochemical compounds [2]. Herbal medicines are becoming increasingly popular due to their fewer side effects, better patient tolerability, lower cost compared with synthetic drugs, and their long history of use has led to greater acceptance [9]. A recent study conducted in 2023 reviewed the usefulness of plant extracts and active compounds to overcome the MDR *A. baumannii* burden [6].

*Rhus coriaria* L. is a typical plant that grows wild in a wide region from the Canary Islands on the Mediterranean coast to Iran and Afghanistan [10]. It is a very popular spice in Mediterranean and Arabian countries, including Syria, Lebanon, Palestine, and Jordan, obtained by grinding the dried fruits [11]. The antifungal, anti‐inflammatory, antitumor, fibrotic, and antioxidant effects of its extract have been proven [12]. Its antimicrobial effect against both Gram‐positive and Gram‐negative bacteria was also confirmed [13]. Additionally, aqueous extracts of *R. coriaria* showed interesting and potent growth repressive effects against MDR *Staphylococcus aureus* strains both in vitro and in vivo [14].

Due to the great pharmaceutical interest in *R. coriaria*, this study investigates the antibacterial and antioxidant activity of this plant against CRAB and may help to identify reliable candidates for drug development against the growing *A. baumannii* threats.

## 2. Methods

### 2.1. Bacterial Isolates

In this study, a total of 10 CRAB isolates were collected from different patients admitted to multiple hospitals between October and December 2022. Sources of isolation include urinary catheter, sputum, wound, and deep tracheal aspiration. The disk diffusion method was performed in the hospital to elucidate the resistance profile against several antimicrobials, where all the bacteria were sensitive only to colistin.

### 2.2. Molecular Characterization

DNA extraction and clean‐up for whole‐genome sequencing, library preparation, sequencing, and bioinformatic analysis were all performed at a collaborating institution.

### 2.3. Plant Extraction

*R. coriaria* was purchased dried, pulverized, and sterile from the market. A total of 10% aqueous extracts of the *R. coriaria* were prepared by adding 10 g of the pulverized seeds to 100 mL of boiling distilled water for 5 min, and then they were left for 30 min to simmer. The extract was then filtered using a sterile muslin cloth. The filtrate was then passed on Whatman No.1 filter paper to eliminate small particles. After that, the filtrate was oven‐dried at 60°C [15]. The water extraction was chosen for many reasons: first, to target water‐soluble bioactive compounds; second, because it is consistent with the traditional water‐based preparation of *R*. *coriaria*; and finally due to its simplicity and safety as an extraction method.

The precipitate was suspended in distilled water to a final concentration of 100 mg/mL, and then aliquoted and kept in the refrigerator at −20°C.

### 2.4. Phytochemical Screening

A qualitative phytochemical examination of *R. coriaria* aqueous extract was performed for the detection of flavonoids, saponins, diterpenes, phenols, glycosides, tannins, and alkaloids, according to the standard protocols described by Vimalkumar et al. [16], Sudarshan et al. [17], and Auwal et al. [18].

### 2.5. Antioxidant Activity

The free radical scavenging antioxidant (RSA) of the seed extract of *R*. *coriaria* L. was tested using a 1,1‐diphenyl‐2‐picryl hydrazyl (DPPH) technique [19]. A 0.6‐mM solution of DPPH in methanol was used as a reference that gave an absorbance of 1.2195 at 517 nm. Three milliliter DPPH was combined with 100 *μ*L of different concentrations of the extract (0.375, 0.75, 1.5, and 3 mg/mL). The tubes were subsequently placed in darkness for 30 min. The absorbance was determined at 517 nm. The IC50 value was calculated using nonlinear regression analysis of the dose–response curve based on all tested concentrations. The percentage of RSA was calculated: *%* = ([A control–A sample]/A control) × 100.

### 2.6. Hemolytic Activity

In vitro, the hemolytic activity of *R*. *coriaria* L. extract was performed following the method of Malagoli [20]. One milliliter of fresh human blood was washed three to five times with 1× phosphate buffer saline (PBS). The supernatant was discarded after centrifugation at 2500 rpm for 10 min. Blood cells (1 mL) were then resuspended in 1× PBS (49 mL) to give a suspension of 2%. Next, 0.2 mL of erythrocyte suspension was added to an equal volume of various extract concentrations (0.375, 0.75, 1.5, and 3 mg/mL). After incubating the mixture at room temperature for 30 min, it was centrifuged at 1500 rpm for 10 min, and the absorbance of the supernatant was then determined at 540 nm. The blank control consisted of PBS mixed with blood, whereas the positive control consisted of blood with 1% SDS, where complete hemolysis is expected. The experiment was conducted thrice at every concentration. The hemolysis percentage for each sample was calculated as (Abs sample/Abs control) × 100 [21].

### 2.7. Antibacterial Activity

#### 2.7.1. Minimum Inhibitory Concentration (MIC) and Minimum Bactericidal Concentration (MBC)

The MIC values of *R. coriaria* were established against all CRABs using the microwell dilution method [22]. The test was conducted in sterile 96‐well microtiter plates by combining 95 *μ*L of nutrient broth and 5‐*μ*L bacterial suspension in each well. Then, 100 *μ*L of *R. coriaria* extracts was serially diluted to obtain concentrations ranging from 3 to 0.012 mg/mL. Plates were incubated at 37°C for 18–24 h. The absorbance was measured at 595 nm.

Following the MIC assay, MBCs were determined by spreading 10 *μ*L from each well on Mueller–Hinton agar (HiMedia, India) plates, then incubated at 37°C for 18–24 h [23].

#### 2.7.2. Time‐ and Dose‐Dependent Growth Inhibitory Activity

Growth of CRAB isolates in contact with *R. coriaria* extract was assessed by calculating the change in the optical density (OD) of the cells grown [22]. Ninety microliters of sterile nutrient broth and 10 *μ*L of bacterial inoculum with a density of approximately 10^5^ CFU/mL were added to each well of a sterile 96‐well microplate. Next, varying concentrations (2× MIC, MIC, and 0.5× MIC) of the extract (100 *μ*L) were added to each well based on the MIC results. The microplates were incubated at 37°C, and the OD was measured at 570 nm at different time intervals (0, 1, 2, 4, 6, 18, and 24 h). The experiments were replicated three times.

#### 2.7.3. Antibiofilm Activity (Inhibition and Destruction of Bacterial Biofilm)

The inhibition of biofilm formation by the aqueous extract was determined according to the method of Olawuwo et al. [24]. Two steps were conducted in the process of biofilm growth: inhibiting biofilm attachment (*T*0) and destroying the biofilm formed in 24 h (*T*24) at distinctive extract concentrations (2MIC, MIC, and 0.5MIC). In the *T*0 study, 100 *μ*L of the bacterial inoculum (10^6^ CFU/ml) was added to sterile flat‐bottomed microtiter plates, followed by 100 *μ*L of extract. The plates were then incubated at 37°C for 24 h without shaking. For the *T*24 study, before adding the extract, the bacterial inoculum was incubated for 24 h to allow biofilm formation. Bacteria and distilled water were used as control (to match and allow proper comparison with treated wells); an additional control with sterile broth and sterile broth was included to confirm normal bacterial growth. After 24 h of incubation, the plates were carefully rinsed thrice with autoclaved distilled water to eliminate unattached or loose cells. Then, it was air‐dried for 5 min and oven‐dried at 60°C for 45 min. The adhered bacterial cells were then dyed with 1% crystal violet (CV) for 15 min at room temperature. Excess CV was washed out with autoclaved distilled water, the bound bacterial cells stained with CV were resolubilized in 120 *μ*L of 95% ethanol, and their absorbance was determined at 595 nm. The inhibition and destruction percentages were calculated as ([OD of control–OD of treated]/OD of control) × 100.

### 2.8. Statistical Analysis

Experiments were performed in triplicate. GraphPad Prism Version 10.1.1 (270) was used to analyze the data and draw all graphs. One‐way ANOVA and *t*‐test were used to calculate the statistically significant differences between values. A *p* value of *< 0.05* was considered significant.

## 3. Results

### 3.1. Whole‐Genome Sequencing Analysis

**Figure 1:**
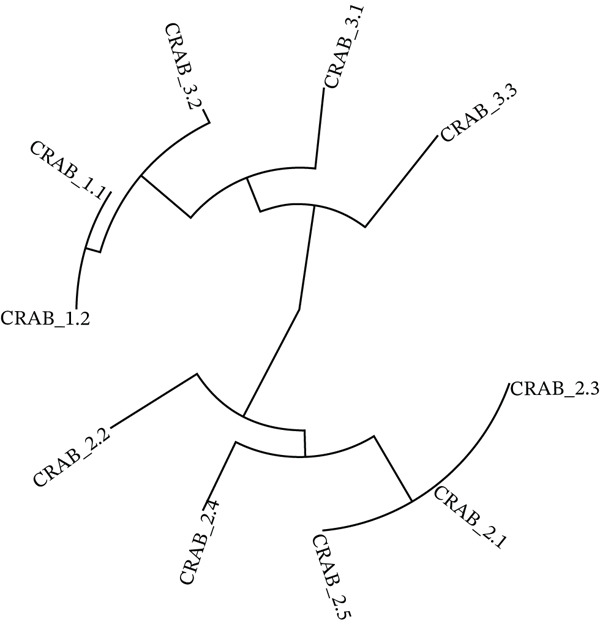
Phylogenetic tree of the different CRAB isolates.

The genomic sequence of the 10 CRAB isolates was uploaded to the National Center for Biotechnology Information NCBI site (https://www.ncbi.nlm.nih.gov) under the accession numbers mentioned in Table 1. The phylogenetic tree of different CRAB isolates based on whole‐genome sequencing is shown in Figure 1.

**Table 1:** Study isolates, accession numbers, and specimen type of CRAB isolates.

Sample	Accession number	Specimen type
CRAB 1.1	SAMN39584154	Urine
CRAB 1.2	SAMN39584155	Urine
CRAB 2.1	SAMN39584156	DTA
CRAB 2.2	SAMN39584157	Urine
CRAB 2.3	SAMN39584158	Sputum
CRAB 2.4	SAMN39584159	DTA
CRAB 2.5	SAMN39584160	DTA
CRAB 3.1	SAMN39584161	Wound
CRAB 3.2	SAMN39584162	Wound
CRAB 3.3	SAMN39584163	Wound

Abbreviations: CRAB, carbapenem‐resistant *Acinetobacter baumannii*; SAMN, sample number.

### 3.2. AMR Gene Analysis

Table S1 summarizes all the AMR genes detected via whole‐genome sequencing. Seven out of 39 genes mediated resistance against carbapenems in the CRAB isolates, including *bla*_OXA-66_, *bla*_OXA-23,_ and *adeJ* in all isolates, *adeI* and *adeK* in 90%, *adeN* in 50%, and *bla*_OXA-72_ in 40% of isolates.

### 3.3. Phytochemical Analysis

Qualitative phytochemical analysis of *R. coriaria* extract was carried out for the evaluation of the presence of different phytochemicals like flavonoids, saponins, diterpenes, phenols, glycosides, tannins, and alkaloids. The extracts showed the presence of all tested phytochemicals, except saponins.

### 3.4. Antioxidant Property of *R*. *coriaria*

The DPPH assay assesses the level of antioxidants. DPPH is a stable free radical, which decolorizes in the presence of antioxidants. Deviations in color, shown by a decrease in the absorbance of DPPH radicals, demonstrate that the antioxidants are present. *R. coriaria* extract possesses significant antioxidant activities as demonstrated by the inhibition percentages calculated at several concentrations ranging from 0.375 to 3 mg/mL (Table 2). Remarkably, scavenging activities of the extract were concentration‐dependent, with a maximum effect seen at 3 mg/mL with a 90.69% inhibition. This activity was comparable with the antioxidant capacity of ascorbic acid (91.66%) at the same concentration. The half‐maximum inhibitory concentration (IC50) obtained from *R. coriaria* extract and ascorbic acid was 0.342 and 0.056 mg/mL, respectively. Although the extract exhibited high antioxidant activity at higher concentrations, it showed lower potency than ascorbic acid, as indicated by its higher IC50 value. Notably, the % scavenging activity at 0.375 mg/mL was 49.887%, which is close to 50% inhibition.

**Table 2:** Antioxidant scavenging potential of *R. coriaria* extract and ascorbic acid using the DPPH assay.

	Concentration (mg/mL)	% scavenging activity	IC50 (mg/mL)
*R. coriaria* extract	0.375	49.87	0.342
	0.75	74.3	
	1.5	79.65	
	3	90.69	

Ascorbic acid (standard)	0.375	81.35	0.056
	0.75	88.87	
	1.5	91.09	
	3	91.66	

### 3.5. Hemolytic Activity

The ability of *R. coriaria* extract to induce hemolysis of human erythrocytes was assessed to evaluate its toxicity at different concentrations (0.375, 0.75, 1.5, and 3 mg/mL). The percentages of hemolysis were very low, ranging from 0.37% to 1.3% at concentrations of 0.375–3 mg/mL, respectively, implying its safety (Table 3).

**Table 3:** Hemolytic activity of *R. coriaria* extract compared with the positive control SDS.

Concentration (mg/mL)	% of Hemolytic activity
Control (SDS)	100%
0.375 mg/mL	0.37%
0.75 mg/mL	0.49%
1.5 mg/mL	0.65%
3 mg/mL	1.3%

### 3.6. Antibacterial Activity

#### 3.6.1. MIC and MBC Assays

The antibacterial efficacy of *R. coriaria* extract was evaluated using MIC and MBC assays. The results indicated an antimicrobial effect against all CRAB isolates with similar MIC and MBC values of 0.75 mg/mL.

#### 3.6.2. Time‐ and Dose‐Dependent Activity

**Figure 2:**
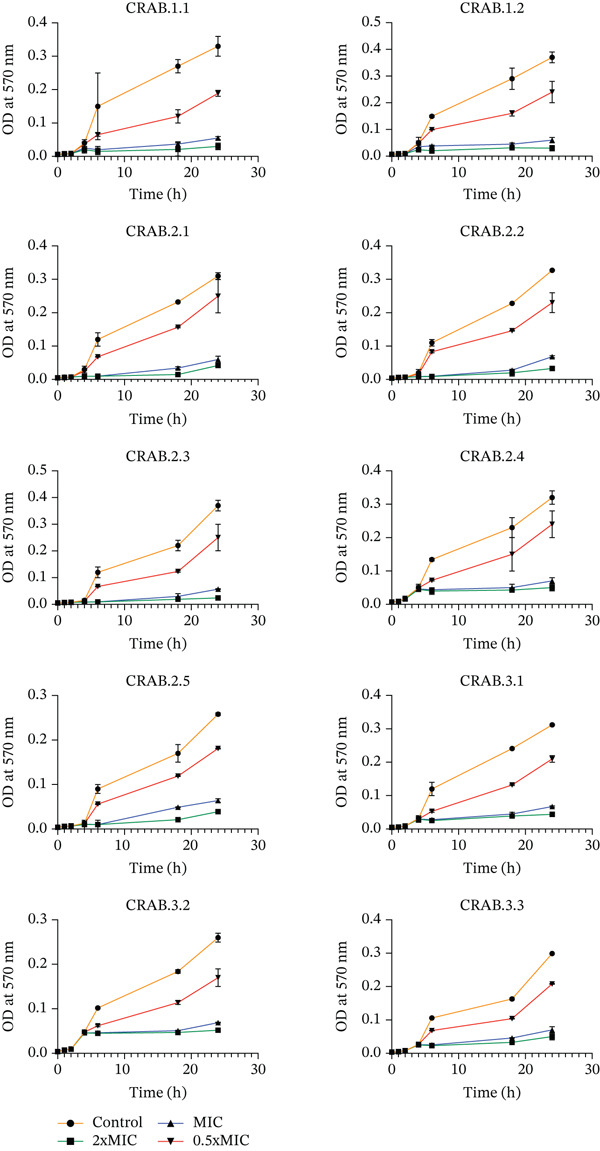
Time‐dependent growth inhibition curves of 10 CRAB isolates against different concentrations of *R. coriaria*, determined by the microplate method, and measured by optical density. Data are expressed as the mean ± SD of three different determinations.

Using the time‐dependent growth inhibition assay, *R. coriaria* exhibited a time‐ and dose‐dependent antibacterial effect on the 10 CRAB isolates. Three different concentrations of *R. coriaria* extracts at 0, 1, 2, 4, 6, 18, and 24 h were assessed as shown in Figure 2. Notably, the data represent bacterial turbidity and do not distinguish between viable and nonviable cells.

An overall decrease in bacterial growth over 24 h, at the three different concentrations, and in all tested bacteria compared with the control was detected. At 0–4 h, all CRAB isolates exhibited a rise in bacterial growth at all concentrations tested. However, a significant (*p* value < 0.0001) rapid decline in growth was observed after 6 h of treatment at 2× MIC and 1× MIC. After 6 h and up until 24 h, at 2× MIC and 1× MIC, a significant reduction in growth was observed compared with the control.

At 0.5× MIC, a significant steady increase in growth was observed up to 24 h, yet it was lower than the control.

Generally, the 2× MIC followed by 1xMIC concentrations were more effective than 0.5× MIC against all tested CRAB isolates, with no difference detected between the different isolates.

#### 3.6.3. Antibiofilm assay

**Figure 3:**
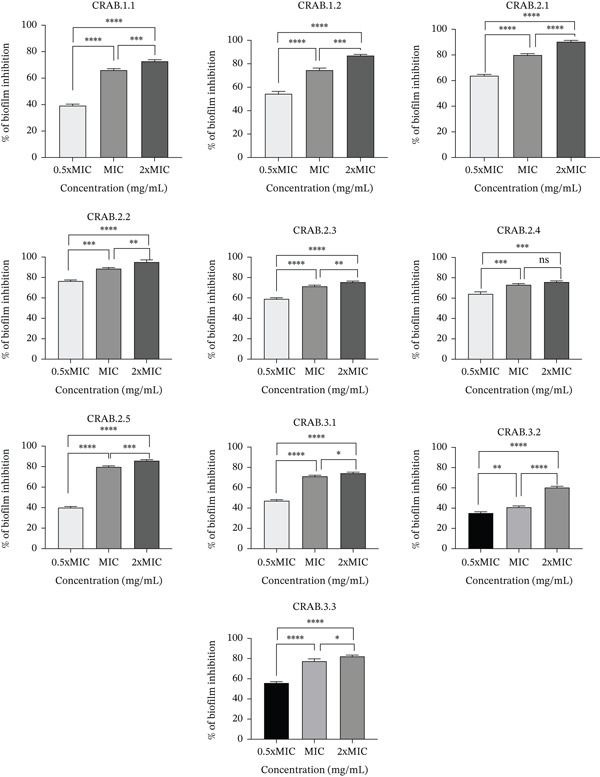
% Inhibition of the 10 CRAB biofilms by different concentrations of *R. coriaria* extract. Data are expressed as the mean ± SD of the triplicate experiment.  ^∗^, *p* ≤ 0.05,  ^∗∗^, *p* ≤ 0.01,  ^∗∗∗^, *p* ≤ 0.001;  ^∗∗∗∗^, *p* ≤ 0.0001.

**Figure 4:**
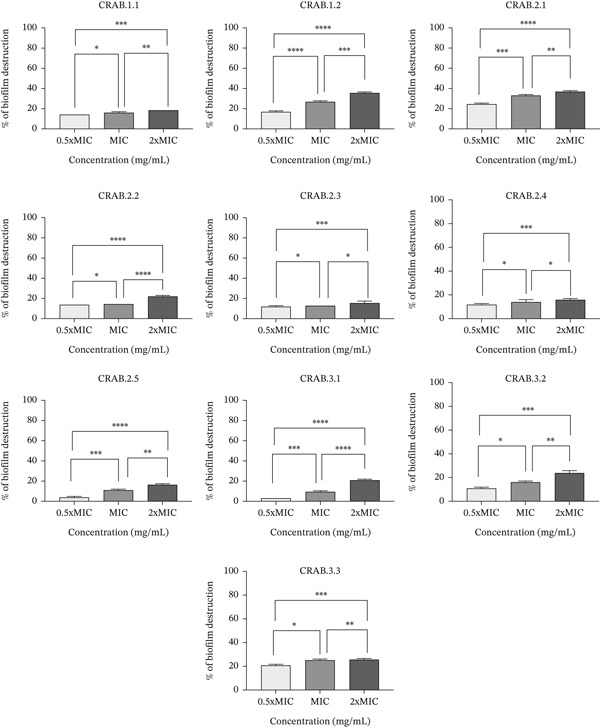
% Destruction of the 10 CRAB biofilms by different concentrations of *R. coriaria* extract. Data are expressed as the mean ± SD of the triplicate experiment.  ^∗^, *p* ≤ 0.05,  ^∗∗^, *p* ≤ 0.01,  ^∗∗∗^, *p* ≤ 0.001;  ^∗∗∗∗^, *p* ≤ 0.0001.

The potential of *R. coriaria* extract to prevent biofilm formation and destruction, measured by CV staining, is illustrated in Figures 3 and 4, respectively. The concentration range used in this assay was defined for all bacteria according to the MIC value.

*R. coriaria* extract significantly inhibited biofilm formation (*p* value < 0.0001), with inhibitory percentages ranging from 60.63% (in CRAB 3.2) up to 95.22% (in CRAB 2.2) at 2× MIC; from 41.17% (CRAB 3.2) reaching 88.7% (CRAB 2.2) at MIC; and from 35.44% (CRAB 3.2) up to 76.61% (CRAB 2.2) at 0.5× MIC. However, the extract possessed low destructive activity on preformed biofilms, with destruction percentages ranging from 15.93% (CRAB 2.4) to 35.52% (CRAB 1.2) at 2× MIC; from 9.44% (CRAB 3.1) to 33.09% (CRAB 2.1) at MIC, and from 3.14% (CRAB 3.1) to 20.76% (CRAB 3.3) at 0.5× MIC.

The best inhibition of biofilm formation and dissolution of preformed biofilms occurred at 2× MIC. Table S2 summarizes the statistically significant differences between the 10 isolates at this concentration.

## 4. Discussion

*A*. *baumannii* is a pleomorphic, aerobic, Gram‐negative coccobacillus [25]. It is an emerging pathogen associated with healthcare‐associated infections and is usually considered to be of low virulence among healthy individuals [26]. However, a wide extent of natural stressors may be confronted by the genetic adaptability of bacteria. Consequently, bacteria living near antimicrobial‐producing organisms have developed survival mechanisms that allow them to live despite the antimicrobial presence [27]. Likewise, *A. baumannii* is a bacterium that has developed various resistance mechanisms to several antibiotics, leading to the development of MDR phenotype, including resistance to the last resort antimicrobial “carbapenem”, thus highlighting the requirement for strategies to develop novel antibiotics [28, 29].

Recognizing acquired AMR genes in *A. baumannii* is crucial in comprehending the processes involved in gaining and evolving AMR [30]. The current study confirmed the presence of 39 different AMR genes mediated resistance to different drug classes, disinfectants, and antiseptics. Moreover, seven of these genes were identified as carbapenem resistance–associated genes, including *adeI, adeJ, adeK, adeN*, *bla*_OXA-23_, *bla*_OXA-66_, and *bla*_OXA-72_. Consequently, *bla*_OXA-23_ is considered to be one of the most important carbapenemases in *A. baumannii* [31]. In a study conducted in 2023, the phytochemical vebonol present in *R. coriaria* showed a high binding affinity (−12.67 kcal/mol) to *bla*_OXA-23_, indicating a high inhibitory activity of this plant against the *bla*_OXA-23_ gene [32]. Importantly, despite the presence of these resistance genes, the aqueous extract of *R. coriaria* in this study demonstrated antimicrobial activity against the clinical isolates, suggesting that its effect may not be compromised by classical carbapenem‐resistance mechanisms such as *β*‐lactamase production and efflux pump activity. This supports the hypothesis that plant‐derived phytochemicals may act through alternative antibacterial pathways, including membrane disruption or induction of cellular stress, which are less affected by resistance genes.

The recent focus on plant‐based antimicrobials has renewed attention to the traditional medicinal uses of *R. coriaria*. Traditionally, this plant has been used in Middle Eastern and Mediterranean areas for its therapeutic properties, including antimicrobial, anti‐inflammatory, and antioxidant benefits. In traditional medicine, *R. coriaria* has been used to address gastrointestinal disorders, wound infections, ulcers, and inflammatory diseases, indicating the existence of bioactive compounds with broad pharmacological potential [33, 34]. These ethnopharmacological uses provide adequate rationale for the extraction method used in this study, particularly aqueous extraction, which reflects traditional preparation methods. Furthermore, *R. coriaria* is rich in diverse phytochemicals, including flavonoids, tannins, and phenolic compounds, which are known to contribute to its potent antioxidant and antibacterial properties [35]. The documented antimicrobial activity of this plant supports its investigation against MDR pathogens such as CRAB. Therefore, combining traditional knowledge with microbiological techniques not only confirms the plant’s importance but also strengthens the scientific basis for investigating its antioxidant and antibacterial properties.

Research findings on the biological activity of *R. coriaria* have designated antioxidant, anti‐inflammatory, anticancer, neuroprotective, and hepatoprotective outcomes due to its phytochemical components [33, 36, 37]. Phytochemicals identified in the *R. coriaria* extract may enhance its antibacterial properties through multiple mechanisms. These compounds are increasingly recognized for their ability to act as direct antimicrobial agents or as possible adjuvants that enhance antibiotic efficacy. Their mechanisms of action include alteration of membrane permeability, inhibition of efflux pumps, and interference with vital cellular processes crucial for bacterial survival, especially in resistant Gram‐negative pathogens [38]. In particular, tannin‐rich plant extracts have been reported to exert strong antibacterial effects against *Pseudomonas aeruginosa*, in which Ł omanowska et al. [39] demonstrated that tannin, from *Rhus typhina*, exhibits antibacterial activity against this bacterium through disruption of membrane integrity via interactions with lipids and proteins, leading to increased permeability and cellular dysfunction. They also reported that tannins can stabilize complexes with membrane proteins, resulting in structural and functional alterations that compromise bacterial viability and resistance mechanisms. Additionally, the choice of extraction solvent may affect the phytochemical profile and biological activity of *R. coriaria*, as different solvents extract different phytochemicals, which could explain differences between studies [37].

In this study, the DPPH‐free RSA of the aqueous extract of *R. coriaria* was determined as 90.69% at a concentration of 3 mg/mL. However, in a study reported in 2022, DPPH‐free scavenging activities of ethanolic *R. coriaria* extract were determined as 70.78% at a concentration of 100 *μ*g/mL [36]. Moreover, in an Iranian study, the antioxidant activity of *R. coriaria* aqueous extract by DPPH was found to be approximately similar to this study, with a percentage of 37%, 90%, and 96% at concentrations 1, 2, and 4 mg/mL, respectively [40]. The hemolytic activity of *R. coriaria* extract was screened against RBCs (red blood cells). In this assay, erythrocytes were exposed to *Rhus* extract, and initiation of hemoglobin discharge, destruction, modifications of RBCs membrane structure, and lysis are the cytotoxicity markers [41]. The hemolytic test was evaluated since the destruction of human erythrocytes will prevent the utilization of *R*. *coriaria* extract. Results indicated that the extract did not affect the RBCs, in which the hemolytic percentage at a concentration of 3 mg/mL was only 1.3%, with a dose‐dependent increase in hemolytic activity. To the best of our knowledge, the hemolytic activity of *R. coriaria* has not been evaluated before. A study done by Karneeb et al. showed a high (34%) and moderate (16%) hemolytic activity of sage essential oils (1.77 mg/mL) and methanolic extract (2 mg/mL), respectively. Although the hemolytic activity of sage aqueous extract agreed with our results, where no hemolysis was shown at a concentration range of 0.625–5 mg/mL [42].

Medicinal plants may represent a potential approach for managing resistant bacteria due to their diverse bioactive compounds; however, their safety and efficacy depend on factors like dosage, preparation, and specific constituents [43]. The results of this study showed that the *R. coriaria* L. aqueous extract inhibited the growth of the tested CRAB isolates. The antibacterial effect of *R. coriaria* against 10 CRAB isolates was evaluated, and it exhibited an inhibitory effect against all CRABs with MIC and MBC values of 0.75 mg/mL. A previous study likewise demonstrated the activity of this extract against five common oral Gram‐positive bacteria, including *Streptococcus mutans, Streptococcus sanguinis, Streptococcus sobrinus*, *Streptococcus salvanius*, and *Enterococcus faecalis,* with MIC range of 0.781–1.562 mg/mL, but an MBC range of 3.125–25 mg/mL [44]. Another study done in 2021 showed an antibacterial effect of this extract also against Gram‐negative bacteria *Klebsiella pneumoniae, Escherichia coli,* and *Shigella species* with MIC values ranging from 0.125 to 0.5 mg/mL and MBC values ranging from 0.25 to 1 mg/mL [45]. However, a study done by Morombaye et al. showed that the antimicrobial activity of *Nepeta cataria* and *Basella alba* methanolic extracts against resistant *A. baumannii* was very low compared with this study, with a MIC value of 60 mg/mL [9].

The time and dose‐dependent effect of *R. coriaria* extract was demonstrated by a time‐dependent growth inhibition test. Wherein, results showed that all CRABs went through a time‐dependent growth phase at 0–4 h, and then were inhibited after 6 h of treatment, where the growth rate was decreased compared with the control group. Moreover, at 2× MIC and 1× MIC, bacterial growth was inhibited more than at 0.5× MIC, proving the extract′s concentration dependency. A study done by Hao et al., indicated that at both MIC (1.04 mg/mL) and 2× MIC of *Litsea cubeba* essential oil, a complete inhibition of *A. baumannii* was detected [46]. Moreover, *Ocimum sanctum*–based silver nanoparticles had also both time and dose‐dependent action against resistant *A. baumannii* [47].

Biofilm formation remains a global public health issue, particularly in terms of nosocomial infections and foodborne sickness [48]. Furthermore, the biofilm inhibition activity of *R. coriaria* extract has been rarely examined. Results of the current study showed good inhibitory activity of biofilm formation of this extract in all CRAB isolates, reaching 95.22% at 2× MIC. However, this extract has less destructive activity. One of the earlier findings confirmed the antibiofilm activity of *R. coriaria* against Gram‐positive bacteria *Listeria monocytogenes* (60%) and *S*. *aureus* (80%), more than Gram‐negative bacteria *E. coli* (25%) and *P*. *aeruginosa* (20%) [48]. Instead, in a study done by Jayathilaka et al., the assays showed that Octominin peptide not only prevented biofilm formation but also effectively destroyed biofilms produced by *A. baumannii* [49]. The reduced efficacy of the extract against established biofilms, as highlighted in this study, may be due to the structural and physiological features of preformed biofilm. At this stage, bacterial cells are embedded within a dense extracellular polymeric substance (EPS) matrix composed of polysaccharides, proteins, and extracellular DNA, thereby limiting the penetration and diffusion of antimicrobial agents. Moreover, cells within mature biofilms tend to demonstrate decreased metabolic activity and altered gene expression, which further reduces their susceptibility to antimicrobial substances [50, 51]. Conversely, the significant inhibitory effect of the *R. coriaria* extract on biofilm formation (up to 95%) indicated that it is particularly potent during the initial stages of biofilm development, when bacterial cells are more accessible and metabolically active. This highlights a potential advantage of the extract as a preventive agent, capable of interfering with initial bacterial adhesion and biofilm establishment, which is a critical step in the pathogenesis of *A*. *baumannii* infections.

## 5. Conclusion

In this study, *R*. *coriaria* aqueous extract demonstrates promising in vitro antimicrobial activity against CRAB isolates. Since this extract has revealed antibacterial activity against different CRAB strains. Furthermore, it also exhibited a good antioxidant capacity, without any hemolytic action against RBCs. However, additional research is required to elucidate the plant′s active compounds using HPLC or GC‐MS and its mechanism of action. Along with toxicity and antibacterial studies using animal models. Additionally, further investigations, including combination studies with conventional antibiotics and isolation of active compounds, are necessary to determine whether *R. coriaria* extract could be developed as an effective antibiotic or adjuvant treatment.

## Author Contributions

Material preparation, data collection, and analysis were performed by Israa Assaf and Jamilah Borjac. Formal analysis for the genomic part was done by Ziad Jabbour and Antoine Abou Fayyad. The first draft of the manuscript was written by Israa Assaf. Ziad Jabbour and Jamila Borjac corrected all versions of the manuscript. All authors contributed to the study conception and design.

## Funding

No funding was received for this manuscript.

## Disclosure

All the authors have taken accountability for the complete content of this manuscript and consented to its submission.

## Ethics Statement

Permission letter was obtained from the hospitals before taking the bacterial samples from their laboratories.

## Conflicts of Interest

The authors declare no conflicts of interest.

## Supporting Information

Additional supporting information can be found online in the Supporting Information section. Additional Supporting Information tables can be found in the Supporting Information section.

## Supporting information


**Supporting Information 1** Table S1: Table S1 includes the antimicrobial resistance genes found in the 10 sequenced CRAB isolates.

**Supporting Information 2** Table S2. Table S2 includes the statistically significant differences in biofilm inhibition between the 10 isolates.

## Data Availability

The data that support the findings of this study are available from the corresponding author upon reasonable request.
