# Supplementary material for: Exploring the Antibacterial and Antioxidant Effects of Rhus coriaria L. Aqueous Extract Against Carbapenem‐Resistant Acinetobacter baumannii
Source: Int J Microbiol. 2026 Apr 30;2026:5238068. doi: 10.1155/ijm/5238068 (PMC13130849; doi:10.1155/ijm/5238068)
Supplement: Supplementary file 1 — Supporting Information 1 Table S1: Table S1 includes the antimicrobial resistance genes found in the 10 sequenced CRAB isolates. [file IJM-2026-5238068-s002.docx]

**Supplemental Table 1**

**Table 1** Antimicrobial resistance genes found the 10 sequenced CRAB isolates.

|  | CRAB_1.1 | CRAB_1.2 | CRAB_2.1 | CRAB_2.2 | CRAB_2.3 | CRAB_2.4 | CRAB_2.5 | CRAB_3.1 | CRAB_3.2 | CRAB_3.3 |
| --- | --- | --- | --- | --- | --- | --- | --- | --- | --- | --- |
| AbaF | + | + | + | + | + | + | + | + | + | + |
| AbaQ | + | + | + | - | + | + | + | + | + | + |
| AmvA | + | + | - | + | + | + | + | + | + | + |
| gyrA | + | + | + | + | + | + | + | + | + | + |
| parC | + | + | + | + | + | + | + | + | + | + |
| abeM | + | + | + | + | + | + | + | + | + | + |
| abeS | + | + | + | + | + | + | + | + | + | + |
| LpsB | + | + | + | + | + | + | + | + | + | + |
| adeA | + | + | + | + | + | + | + | + | + | + |
| adeB | + | + | + | + | + | + | + | + | + | + |
| adeC | + | + | + | + | + | + | + | + | + | + |
| adeF | + | + | + | + | + | + | + | + | + | + |
| adeG | + | + | + | + | + | - | + | + | + | + |
| adeH | + | + | + | - | + | - | + | + | + | + |
| adeI | + | - | + | + | + | + | + | + | + | + |
| adeJ | + | + | + | + | + | + | + | + | + | + |
| adeK | + | + | - | + | + | + | + | + | + | + |
| adeL | + | + | + | + | + | + | + | + | + | + |
| adeN | - | - | + | + | + | + | + | - | - | - |
| adeS | + | + | + | - | + | + | + | + | + | + |
| adeR | + | + | + | + | + | + | + | + | + | + |
| ANT (3”)-IIc | + | + | - | - | - | + | + | + | + | + |
| ADC-30 | - | - | - | - | - | - | - | - | - | + |
| ADC-73 | + | + | + | + | + | + | + | + | + | - |
| tet(B) | + | - | - | - | - | - | - | - | - | - |
| tet(R) | + | + | - | - | - | - | - | + | + | - |
| APH (6)-Id | + | + | - | - | - | - | - | + | + | + |
| APH (3’)-VIa | + | + | - | - | - | - | - | - | - | + |
| APH (3”)-Ib | - | + | + | + | - | - | - | + | + | + |
| APH (3’)-Ia | - | - | - | - | + | + | + | + | - | - |
| Oxa-23 | + | + | + | + | + | + | + | + | + | + |
| Oxa-66 | + | + | + | + | + | + | + | + | + | + |
| Oxa-72 | - | - | + | + | - | + | + | - | - | - |
| rsmA | + | + | - | + | + | + | + | + | + | + |
| msrE | - | - | + | + | - | + | + | + | - | + |
| mphE | - | - | + | + | - | + | + | + | - | + |
| sul-2 | - | - | + | - | + | + | + | - | - | + |
| TEM-1 | - | - | + | + | + | + | + | + | - | - |
| armA | - | - | - | + | - | + | + | + | - | + |

(+) indicates the presence of the gene; (-) indicates the absence of the gene
