# Supplementary material for: Exploring the Antibacterial and Antioxidant Effects of Rhus coriaria L. Aqueous Extract Against Carbapenem‐Resistant Acinetobacter baumannii
Source: Int J Microbiol. 2026 Apr 30;2026:5238068. doi: 10.1155/ijm/5238068 (PMC13130849; doi:10.1155/ijm/5238068)
Supplement: Supplementary file 2 — Supporting Information 2 Table S2. Table S2 includes the statistically significant differences in biofilm inhibition between the 10 isolates. [file IJM-2026-5238068-s001.docx]

**Supplemental Table 2**

**Table 2:** *P-values* of the results of the biofilm inhibition of *Rhus* *coriaria* on the different CRAB.

|  | Biofilm Inhibition | | Biofilm Destruction | |
| --- | --- | --- | --- | --- |
| Samples | *p-value* | Significance | *p-value* | Significance |
| CRAB.1.1 vs. CRAB.1.2 | <0.0001 | **** | <0.0001 | **** |
| CRAB.1.1 vs. CRAB.2.1 | <0.0001 | **** | <0.0001 | **** |
| CRAB.1.1 vs. CRAB.2.2 | <0.0001 | **** | 0.0493 | * |
| CRAB.1.1 vs. CRAB.2.3 | 0.3258 | ns | 0.1412 | ns |
| CRAB.1.1 vs. CRAB.2.4 | 0.1956 | ns | >0.9999 | ns |
| CRAB.1.1 vs. CRAB.2.5 | <0.0001 | **** | 0.5708 | ns |
| CRAB.1.1 vs. CRAB.3.1 | 0.9327 | ns | 0.3166 | ns |
| CRAB.1.1 vs. CRAB.3.2 | <0.0001 | **** | 0.001 | *** |
| CRAB.1.1 vs. CRAB.3.3 | <0.0001 | **** | <0.0001 | **** |
| CRAB.1.2 vs. CRAB.2.1 | 0.0709 | ns | 0.9236 | ns |
| CRAB.1.2 vs. CRAB.2.2 | <0.0001 | **** | <0.0001 | **** |
| CRAB.1.2 vs. CRAB.2.3 | <0.0001 | **** | <0.0001 | **** |
| CRAB.1.2 vs. CRAB.2.4 | <0.0001 | **** | <0.0001 | **** |
| CRAB.1.2 vs. CRAB.2.5 | 0.9809 | ns | <0.0001 | **** |
| CRAB.1.2 vs. CRAB.3.1 | <0.0001 | **** | <0.0001 | **** |
| CRAB.1.2 vs. CRAB.3.2 | <0.0001 | **** | <0.0001 | **** |
| CRAB.1.2 vs. CRAB.3.3 | 0.0079 | ** | <0.0001 | **** |
| CRAB.2.1 vs. CRAB.2.2 | 0.0046 | ** | <0.0001 | **** |
| CRAB.2.1 vs. CRAB.2.3 | <0.0001 | **** | <0.0001 | **** |
| CRAB.2.1 vs. CRAB.2.4 | <0.0001 | **** | <0.0001 | **** |
| CRAB.2.1 vs. CRAB.2.5 | 0.0082 | ** | <0.0001 | **** |
| CRAB.2.1 vs. CRAB.3.1 | <0.0001 | **** | <0.0001 | **** |
| CRAB.2.1 vs. CRAB.3.2 | <0.0001 | **** | <0.0001 | **** |
| CRAB.2.1 vs. CRAB.3.3 | <0.0001 | **** | <0.0001 | **** |
| CRAB.2.2 vs. CRAB.2.3 | <0.0001 | **** | <0.0001 | **** |
| CRAB.2.2 vs. CRAB.2.4 | <0.0001 | **** | 0.0168 | * |
| CRAB.2.2 vs. CRAB.2.5 | <0.0001 | **** | 0.0006 | *** |
| CRAB.2.2 vs. CRAB.3.1 | <0.0001 | **** | 0.9863 | ns |
| CRAB.2.2 vs. CRAB.3.2 | <0.0001 | **** | 0.7146 | ns |
| CRAB.2.2 vs. CRAB.3.3 | <0.0001 | **** | 0.0474 | * |
| CRAB.2.3 vs. CRAB.2.4 | >0.9999 | ns | 0.3309 | ns |
| CRAB.2.3 vs. CRAB.2.5 | <0.0001 | **** | 0.9926 | ns |
| CRAB.2.3 vs. CRAB.3.1 | 0.9704 | ns | 0.0007 | *** |
| CRAB.2.3 vs. CRAB.3.2 | <0.0001 | **** | <0.0001 | **** |
| CRAB.2.3 vs. CRAB.3.3 | <0.0001 | **** | <0.0001 | **** |
| CRAB.2.4 vs. CRAB.2.5 | <0.0001 | **** | 0.8578 | ns |
| CRAB.2.4 vs. CRAB.3.1 | 0.8844 | ns | 0.1338 | ns |
| CRAB.2.4 vs. CRAB.3.2 | <0.0001 | **** | 0.0003 | *** |
| CRAB.2.4 vs. CRAB.3.3 | 0.0001 | *** | <0.0001 | **** |
| CRAB.2.5 vs. CRAB.3.1 | <0.0001 | **** | 0.0055 | ** |
| CRAB.2.5 vs. CRAB.3.2 | <0.0001 | **** | <0.0001 | **** |
| CRAB.2.5 vs. CRAB.3.3 | 0.0760 | ns | <0.0001 | **** |
| CRAB.3.1 vs. CRAB.3.2 | <0.0001 | **** | 0.1837 | ns |
| CRAB.3.1 vs. CRAB.3.3 | <0.0001 | **** | 0.0053 | ** |
| CRAB.3.2 vs. CRAB.3.3 | <0.0001 | **** | 0.7595 | ns |

*CRAB, Carbapenem-resistant Acinetobacter baumannii*

*ns, not significant; *, p* ≤ *0.05, **, p* ≤ *0.01, ***, p* ≤ *0.001; ****, p* ≤ *0.0001*
